# Prognostic Value of ^18^F-FDG PET/CT in Surgical Non-Small Cell Lung Cancer: A Meta-Analysis

**DOI:** 10.1371/journal.pone.0146195

**Published:** 2016-01-04

**Authors:** Jing Liu, Min Dong, Xiaorong Sun, Wenwu Li, Ligang Xing, Jinming Yu

**Affiliations:** 1 Department of Radiation Oncology and Shandong Key Laboratory of Radiation Oncology, Shandong Cancer Hospital and Institute, Jinan, Shandong, China; 2 Department of Oncology, the People’s Hospital of Pingyi County, Pingyi, Shandong, China; 3 Department of Radiology, Shandong Cancer Hospital and Institute, Jinan, Shandong, China; Roswell Park Cancer Institute, UNITED STATES

## Abstract

**Background:**

The identification of surgical non-small cell lung cancer (NSCLC) patients with poor prognosis is a priority in clinical oncology because of their high 5-year mortality. This meta-analysis explored the prognostic value of maximal standardized uptake value (SUV_max_), metabolic tumor volume (MTV) and total lesion glycolysis (TLG) on disease-free survival (DFS) and overall survival (OS) in surgical NSCLC patients.

**Materials and Methods:**

MEDLINE, EMBASE and Cochrane Libraries were systematically searched until August 1, 2015. Prospective or retrospective studies that evaluated the prognostic roles of preoperative 18F-FDG PET/CT with complete DFS and OS data in surgical NSCLC patients were included. The impact of SUV_max_, MTV or TLG on survival was measured using hazard ratios (HR). Sub-group analyses were performed based on disease stage, pathological classification, surgery only and cut-off values.

**Results:**

Thirty-six studies comprised of 5807 patients were included. The combined HRs for DFS were 2.74 (95%CI 2.33–3.24, unadjusted) and 2.43 (95%CI: 1.76–3.36, adjusted) for SUV_max_, 2.27 (95%CI 1.77–2.90, unadjusted) and 2.49 (95%CI 1.23–5.04, adjusted) for MTV, and 2.46 (95%CI 1.91–3.17, unadjusted) and 2.97 (95%CI 1.68–5.28, adjusted) for TLG. The pooled HRs for OS were 2.54 (95%CI 1.86–3.49, unadjusted) and 1.52 (95%CI 1.16–2.00, adjusted) for SUV_max_, 2.07 (95%CI 1.16–3.69, unadjusted) and 1.91 (95%CI 1.13–3.22, adjusted) for MTV, and 2.47 (95%CI 1.38–4.43, unadjusted) and 1.94 (95%CI 1.12–3.33, adjusted) for TLG. Begg’s test detected publication bias, the trim and fill procedure was performed, and similar HRs were obtained. The prognostic role of SUV_max_, MTV and TLG remained similar in the sub-group analyses.

**Conclusions:**

High values of SUV_max_, MTV and TLG predicted a higher risk of recurrence or death in patients with surgical NSCLC. We suggest the use of FDG PET/CT to select patients who are at high risk of disease recurrence or death and may benefit from aggressive treatments.

## Introduction

The application of advanced diagnostic and screening techniques led to the increased detection of early staged non-small cell lung cancers (NSCLC) and improved cures using standard surgery [1]. The 5-year survival after resection of localized NSCLC approaches a modest 50% despite improved surgical techniques and advanced adjuvant therapy [2, 3]. No prognostic factor, except stage and performance status, was definitively established in NSCLC. Accurate markers would be invaluable to stratify patients for adjuvant therapy and predict outcomes.

^18^F-fluorodeoxyglucose (FDG) positron emission tomography/computed tomography (PET/CT) is the standard modality for staging, treatment response monitoring and prognosis prediction for a variety of tumors, including NSCLC [4, 5]. Standardized uptake value (SUV) is a semi-quantitative determination of the normalized concentration of radioactivity, and maximum SUV (SUV_max_) is the most widely applied parameter in clinical practice [6]. Volumetric parameters, including metabolic tumor volume (MTV) and total lesion glycolysis (TLG), were also used recently to reflect disease burden and tumor aggressiveness in NSCLC [4, 7]. Several recent systematic reviews and meta-analyses [8–10] found that SUV was negatively correlated with prognosis in heterogeneous groups of NSCLC patients. Im et al. [11] reported significant prognostic values of MTV and TLG on survival in NSCLC patients. However, the quality of existing studies has not been systematically assessed, and their clinical features have not been fully assessed to further evaluate the potential association between SUV or volumetric parameters and prognosis in surgical NSCLC.

Therefore, we performed a meta-analysis to identify, appraise, and synthesize results from published studies that examined the prognostic value of SUV_max_, MTV and TLG on disease-free survival (DFS) and overall survival (OS) in surgical NSCLC patients.

## Materials and Methods

### Search Strategy and Eligible Criteria

MEDLINE, EMBASE and Cochrane Library were searched and updated through August 1, 2015. The following terms were used: “non-small cell lung cancer” OR “NSCLC” OR “carcinoma, non-small cell lung” AND “^18^F-FDG” OR “fluorodeoxyglucose” OR “PET” OR “positron emission tomography” AND “survival” OR “local control” OR “prognostic” OR “outcome” OR “predict” AND “surgery” OR “resect” OR “operation”. Reviews, case studies, conference abstracts and editorials were excluded.

Two authors independently searched articles and performed an initial screening of identified titles and abstracts. Articles were further reviewed if they reported the prognosis of surgically resected NSCLC patients with pre-operative ^18^F-FDG PET/CT imaging from original data. Full-text articles were used for the second screening. The following inclusion criteria for the meta-analysis were used: (1) prospective or retrospective studies investigating the correlation of FDG uptake with DFS, recurrence-free survival (RFS), and/or OS; (2) pathological stage I-IIIA NSCLC patients who received diagnostic ^18^F-FDG PET/CT scanning before treatments; (3) treated with surgery alone or adjuvant therapy; (4) survival data assessed in detail; and (5) surgical procedures included either full anatomical resections or limited lung resection regardless of whether they were performed via open thoracotomy or video-assisted thoracic surgery. A consensus resolved any discrepancies.

Studies that included patients with small cell lung cancer (SCLC) were eligible if more than 95% of patients had NSCLC. Patients with an advanced stage (IIIB-IV) also accounted for less than 5% of the included studies. Data were partially extracted when only certain sub-group analyses met our inclusion criteria. Studies that included patients who received neoadjuvant therapy were excluded. Only the most recent or complete report was included when the survival results of the same patient population were reported more than once.

### Data Extraction

Data extraction was conducted in agreement with the Preferred Reporting Items for Systematic Reviews and Meta-Analyses (PRISMA) guidance (S1 PRISMA Checklist) [12]. Two investigators independently extracted information, including the first author, publication year, country, study design, sample size, stage, treatment, and survival endpoints. The primary endpoint was DFS, which was measured from the defined starting point in each study to the date of recurrence or first progression. OS was taken as the secondary endpoint and defined as the time from the starting point applied in each study to death.

### Study Quality Control

Three investigators reviewed and scored each article independently using a quality scale (S1 File). Quality assessment included four modified parts based on similar studies: scientific design, the generalizability of the results, data analyses, and PET reports [13–15]. Five items were observed in each part. A point value of 0, 1, or 2 was scored to each item. A consensus was obtained of all investigators present, which ensured the objectivity of the scores and correct interpretation. Final scores are expressed as percentages, and higher values reflect a greater consistence with quality assessment standards. Any article with a final score < 60% was excluded.

### Statistical Analysis

Review Manager statistical software (RevMan, version 5.3) was used. The impact of SUV_max_, MTV and TLG on DFS and OS was measured using hazard ratios (HRs). Survival data were extracted using the methodology suggested by Tierney et al. [16]. Cut-off values of SUV_max_, MTV and TLG and the delineation thresholds applied to MTV and TLG were determined based on the definition applied in each individual study. Unadjusted and adjusted values were extracted for risk measurements. We extracted the HR estimate and 95% confidence intervals (CIs) directly from each study when provided by the authors. *P* values of the log-rank test, number analyzed in each group, and the number of events were extracted to estimate the univariate HR indirectly. Correlations between the quality scores and the number of patients were measured using the Spearman’s rank correlation coefficient.

Heterogeneity was evaluated using Cochrane’s Q test and *I*^*2*^ [17]. *P*<0.05 in Q test was considered significant heterogeneity. An *I*^*2*^ value of 0% indicates no heterogeneity, a value less than 25% indicates low heterogeneity, a value of 25.1–50% indicates moderate heterogeneity, and a value greater than 50% indicates substantial heterogeneity [18]. A fixed effect model was used to calculate the pooled HRs when no, low or moderate heterogeneity was observed. A random effects model was applied when substantial or significant heterogeneity was observed. An HR greater than 1 implied worse survival outcome for patients with high SUV_max_, MTV or TLG, but an HR less than 1 implied a survival benefit for patients with high SUV_max_, MTV or TLG. Sub-group analyses were performed based on histological subtypes, pathological stage, surgery only and cut-off values.

The possibility of publication bias was estimated using visual inspection of a funnel plot. Begg’s test was performed for meta-analysis that included more than 10 studies [19, 20]. We also performed non-parametric “trim and fill” procedures to further estimate the potential influence of publication bias [21, 22]. This procedure calculates a new pooled HR that incorporates hypothetical missing studies.

## Results

### Study Characteristics and Qualitative Assessment

**Fig 1:**
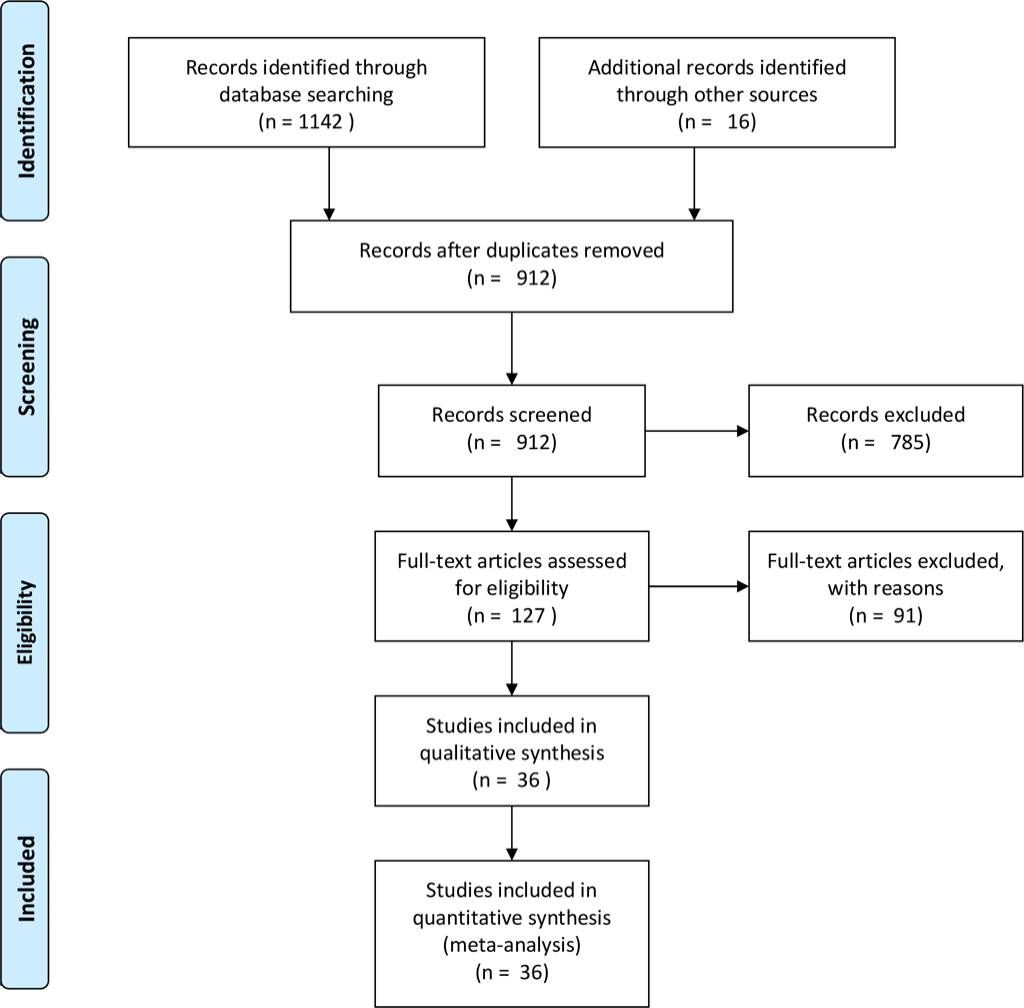
Flowchart of Study Selection.

Thirty-six eligible studies were included in the meta-analysis [23–58] (Fig 1, Tables 1 and 2). Only two studies [31, 37] were prospectively designed. The studies were published between 2000 and 2015, and the sample size varied from 49 to 530 subjects (median 102). Only 5 SCLC [40, 51] patients were mixed into the analysis of 5807 patients. Four studies lacked raw data of stage [23, 29, 40, 49], but the distribution of stages I, II, III and IV were 80.4%, 14.2%, 4.5% (2.7% IIIA, 0.9% IIIB, and 0.9% stage III) and 0.9%, respectively. Table 1 lists PET/CT scans and models. The dose of FDG injected varied from 150 to 666 MBq based on different individual scanning protocols. The time duration before scanning was 40–60 minutes in 28 studies, 81 minutes in 1 study, 90 minutes in 1 study and not reported in 6 studies. SUV_max_ was measured in 34 studies [23–25, 28–58], which normalized values by body weight. MTV was measured in 7 studies [24, 26–29, 52, 53], and TLG was measured in 7 studies [24, 26, 27, 29, 52, 53, 56]. A fixed SUV of 2.5 [27, 28, 52, 56], the gradient segmentation method [29], a 50% of SUV_max_ [24], a 42% of SUV_max_ [53], and mediastinal background SUV_mean_ plus 2 standard deviations [26] were adapted to segment volumes of interest. A minimum *P* value, receiver operating characteristics (ROCs), and median value were applied in most studies to determine cut-off values. Median cut-off points were 5.90 (2.4 to 20) for SUV_max_. The cut-off values of MTV were between 2.95 and 37.34 cm^3^ (median 11.197 for OS and 10.29 for DFS), and TLG values ranged from 9.61 to 407.48 (median 26.739 for OS and 29.221 for DFS).

**Table 1:** Studies Included in Meta-Analysis.

Study	Year	Country	Study design	No. of patients	TNM staging	Treatment	Endpoints	PET scanners	PET parameters	Tumor delineation (thresholds)	Determination of cut-off values	Cut-off values		
												SUV	MTV (cm^3^)	TLG
Cistaro et al. [23]	2013	Italy	R	49	I-II	Surgery	DFS	Discovery ST, GE	SUV_max_		Minimum P value method	9.0		
Melloni et al. [24]	2013	Italy	R	99	I	Surgery	DFS	Discovery STE or Discovery 690, GE; GEMINI-GXL, Philips	SUV_max_ /MTV/TLG	50%SUV_max_	Median	2.73	2.95	9.61
Ohtaka et al. [25]	2013	Japan	R	191	I-IIIA	Surgery	OS/DFS	ECAT EXACT HR+, Siemens	SUV_max_		Median	2.4		
Hyun et al. [26]	2013	Korea	R	529	I-II	Surgery ± adjuvant CT/RT/CRT	OS/DFS	Discovery LS, GE	MTV/TLG	Mediastinal background SUV_mean_ plus 2 standard deviations	ROC curve		16	70
Kim et al. [27]	2012	Korea	R	91	I-IIIA	Surgery ± adjuvant CT	OS/RFS	Gemini, Philips; Biograph40, Siemens	MTV/TLG	SUV2.5	ROC curve		11.613/9.598	13.797/18.762
Lin et al. [28]	2012	Taiwan	R	60	I	Surgery	DFS	Discovery STE, GE	SUV_max_/MTV	SUV2.5	Median	2.5	9.8	
Zhang et al. [29]	2013	China	R	59	I-IV	Surgery	OS	Reveal HD, CTI	SUV_max_ /MTV/TLG	Gradient tumor segmentation	ROC curve	11.59	37.34	407.48
Agarwal et al. [30]	2010	USA	R	363	I-II	Surgery	OS	Discovery DST or Advance, GE	SUV_max_		Median	5.9		
Dooms et al. [31]	2009	Belgium	P	91	I-II	Surgery	OS	931/08/12 or ECAT EXACT 922, CTI-Siemens	SUV_max_		Median	NR		
Goodgame et al. [32]	2008	USA	R	136	I	Surgery	Recurrence rate/OS	ECAT EXACT or Biograph LSO Duo hybrid PET/CT, Siemens; ADAC CPET-Plus, Phillips	SUV_max_		Median	5.5		
Hanin et al. [33]	2008	Belgium	R	96	I-II	Surgery	OS/DFS	ECAT HR+, Siemens	SUV_max_		Median	7.8		
Kim et al. [34]	2011	Korea	R	76	I-II	Surgery	OS/DFS	Gemini, Philips	SUV_max_		ROC curve	6.7/5.9		
Nair et al. [35]	2010	USA	R	75	IA	Surgery	OS	CTI	SUV_max_		Minimum P value method	5		
Um et al. [36]	2009	Korea	R	145	I	Surgery	DFS	Discovery LS, GE	SUV_max_		ROC curve	13.1		
Vesselle et al. [37]	2007	USA	P	103	I-III	Surgery	OS/DFS	PET Advance, GE	SUV_max_		Minimum P value method	7		
Zhang et al. [38]	2007	China	R	82	I-III	Surgery ± adjuvant CT	OS/DFS	Discovery LS, GE	SUV_max_		Median	11		
Bille et al. [39]	2013	UK	R	404	I-IV	Surgery ± adjuvant CT	OS	Discovery ST, GE	SUV_max_		Median	8.6		
Dhital et al. [40]	2000	UK	R	77	I-IIIA	Surgery	OS	ECAT 951/31R, Siemens	SUV_max_		Minimum P value method	20		
Higashi et al. [41]	2002	Japan	R	57	I-III	Surgery	OS/DFS	Headtome IV, Shimazu	SUV_max_		Minimum P value method	5		
Kim et al. [42]	2009	Korea	R	107	I	Surgery	DFS	Allegro, Philips	SUV_max_		Median	2.4		
Ohtsuka et al. [43]	2006	Japan	R	98	I	Surgery	DFS	NR	SUV_max_		ROC curve	3.3		
Stiles et al. [44]	2013	USA	R	530	I-IIIA	Surgery	DFS	NR	SUV_max_		Median	4.8		
Tomita et al. [45]	2012	Japan	R	197	I-III	Surgery	OS	Siemens	SUV_max_		Median	6.6		
Tsutani et al. [46]	2011	Japan	R	176	I-III	Surgery	DFS	Discovery ST16, GE	SUV_max_		ROC curve	3.7/6.95		
van Baardwijk et al. [47]	2007	Netherlands	R	102	I-III	Surgery	OS	931/08/12 or ECAT EXACT 922, CTI-Siemens	SUV_max_		Minimum P value method	8/11		
Downey et al. [48]	2007	UK	R	487	I-IV	Surgery	OS	NR	SUV_max_		Median	5.3		
Koo et al. [49]	2011	Korea	R	75	I-II	Surgery	DFS	Gemini, Philips	SUV_max_		ROC curve	4.5		
Pelosi et al. [50]	2011	Italy	R	153	I-IV	Surgery ± adjuvant CT	OS/DFS	Discovery ST, GE	SUV_max_		Minimum P value method	9		
Shiono et al. [51]	2011	Japan	R	201	I	Surgery ± adjuvant CT	DFS	Discovery LS, GE	SUV_max_		ROC curve	4.7		
Kim et al. [52]	2014	Korea	R	102	I-II	Surgery	DFS	Reveal RT-HiREZ, CTI; Discovery STE, GE	SUV_max_ /MTV/TLG	SUV2.5	ROC curve	6.90	10.78	39.68
Domachevsky et al [53]	2015	Israel	R	181	I-II	Surgery	OS	Discovery STE, GE	SUV_max_ /MTV/TLG	42%SUV_max_	Median	8.2	NR	NR
Ko et al [54]	2015	Taiwan	R	145	IA	Surgery	RFS	Biograph, Siemens	SUV_max_		ROC curve	2.5		
Motono et al [55]	2014	Japan	R	58	I-IIIA	Surgery	DFS	Headtome IV, Shimazu	SUV_max_		NR	NR		
Park et al [56]	2015	Korea	R	248	IA	Surgery	OS	Discovery 600, GE; Biograph TruePoint 40, Siemens	SUV_max_ /TLG	SUV2.5	Maximally selected rank	3.7		13.76
Shimizu et al [57]	2014	Japan	R	84	I-III	Surgery	DFS	Discovery ST, GE	SUV_max_		ROC curve	3.95/9.7		
Yoo et al [58]	2014	Korea	R	80	I-IIA	Surgery	DFS	Biographs; Siemens	SUV_max_		Minimum P value method	4		

**Abbreviations:** T: tumor; N: lymph node; M: metastasis; PET: positron emission tomography; SUV: standardized uptake value; MTV: metabolic tumor volume; TLG: total lesion glycolysis; R: retrospective; P: prospective; DFS: disease-free survival; OS: overall survival; SUV_max_: maximal standardized uptake value; CT: chemotherapy; RT: radiotherapy; CRT: chemoradiotherapy; SUV_mean_: mean standardized uptake value; ROC: receiver operating characteristic; RFS: recurrence-free survival; NR: not reported.

**Table 2:** Clinical Characteristics of Included Studies.

Study Criteria	No. of Studies
Study design	
Prospective design	2
Retrospective design	34
Histology	
Adenocarcinoma only	4
NSCLC	30
NSCLC and SCLC	2
Treatment	
Surgery only	30
Surgery ± adjuvant chemotherapy	5
Surgery ± adjuvant chemotherapy/radiotherapy	1
Prognostic parameters	
SUV_max_	34
MTV	7
TLG	7
Determination of cut-off values	
Minimum P value	8
Receiver operating characteristics curve	12
Median value	14
Others	2
HR reported	
Adjusted	25
Unadjusted	35
Multivariate analysis (with adjustment for)	
Tumor stage	14
Stage	11
Age	9
Gender	6
Histology	6
Lymph node status	5
Differentiation	5
Carcino-embryonic antigen level	4
Follow-up schedule	
Well-planned and described in detail	11
Well-planned but not described in detail	5
Not indicated	20

**Abbreviations:** NSCLC: non-small cell lung cancer; SCLC: small cell lung cancer; SUV_max_: maximal standardized uptake value; MTV: metabolic tumor volume; TLG: total lesion glycolysis; DFS: disease-free survival; OS: overall survival; HR: hazard ratio; PET/CT: positron emission tomography/computed tomography.

Adjusted HRs were determined for 25 studies. Most risk measures were adjusted for tumor size or T stage, stage, age, gender and histology, and other studies were adjusted for lymph node status, differentiation and CEA level.

Twenty-seven studies published complete resection rates as 100%, while the remaining studies did not report rates. Average (mean or median) follow-up duration was given in 29 studies and ranged from 16.6 to 64 months (median 32.0 months). The follow-up design was reported in detail in 11 studies, but it was not indicated in 20 studies.

Attempts to contact the authors to obtain missing information of methodological quality were made when necessary, and the mean quality score was 77.5% (70.0% to 87.5%). Spearman’s correlation coefficient was 0.326 between the quality score and the number of patients (*P* = 0.36).

### Primary Outcome: DFS

**Fig 2:**
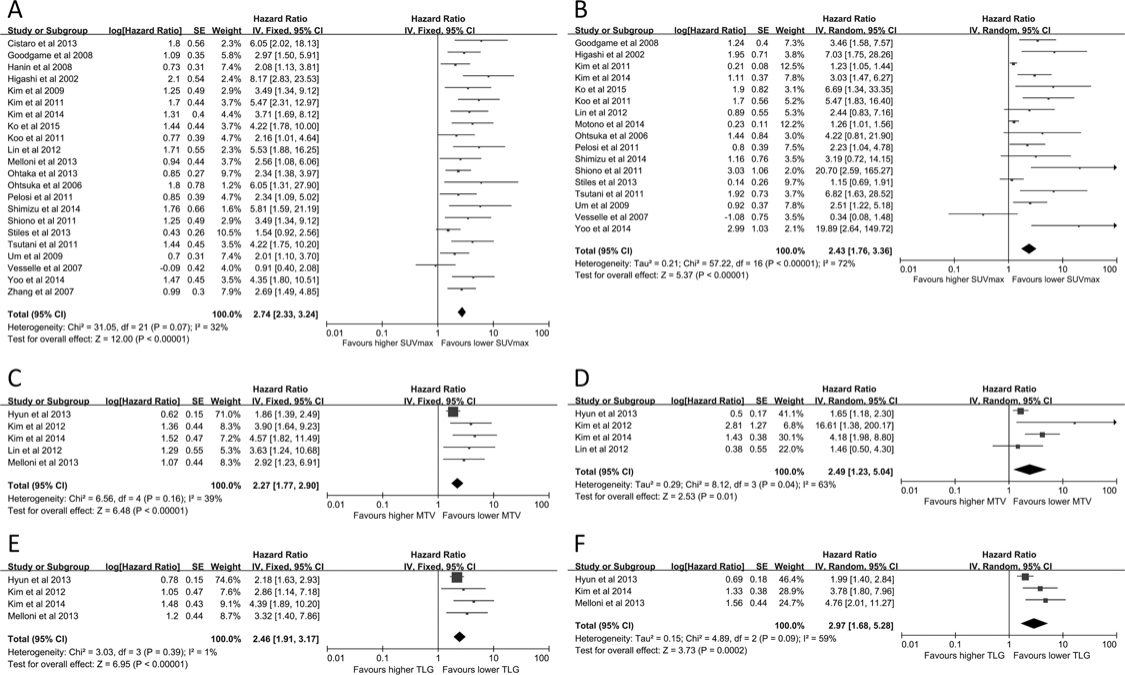
Forest plots of HR for recurrence with SUV_max_ (A, unadjusted; B, adjusted), MTV (C, unadjusted; D, adjusted) and TLG (E, unadjusted; F, adjusted). The Chi^2^ test is a measurement of heterogeneity. *P*<0.05 indicates significant heterogeneity. Squares = individual study point estimates. Horizontal lines = 95% CIs. Rhombus = summarized estimate and its 95% CI. Fixed: fixed effect model. Random: random effect model.

**Fig 3:**
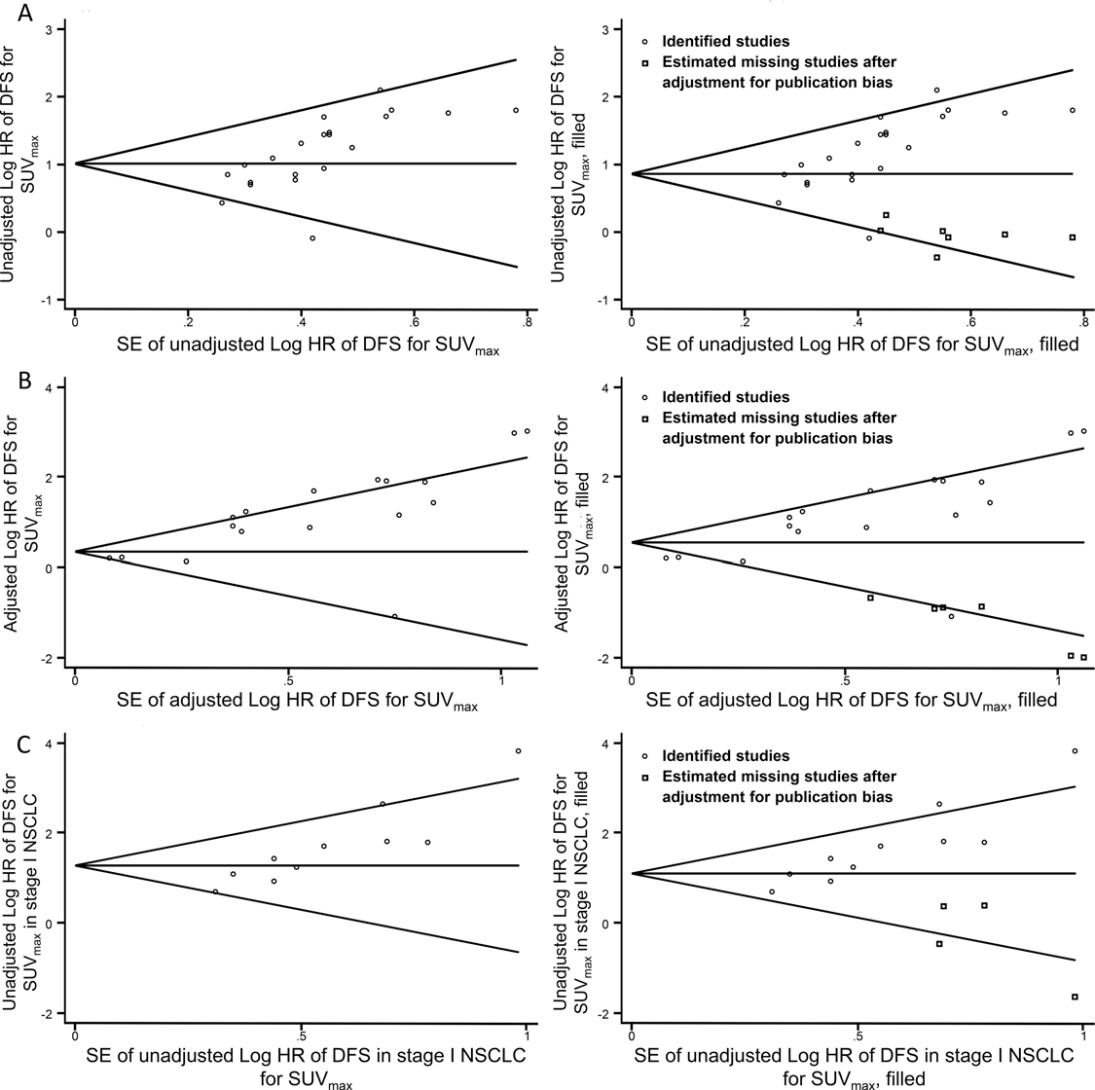
Funnel plots without (left column) and with (right column) trim and fill. The pseudo 95% confidence interval (CI) is computed as part of the analysis that produced the funnel plot and corresponds to the expected 95% CI for a given standard error (SE). HR indicates hazard ratio.

Unadjusted analysis of SUV_max_ and DFS (2845 patients) revealed a combined HR of 2.74 (95%CI: 2.33–3.24, *P* = 0.07, *I*^*2*^ = 32%) (Fig 2A). The funnel plots for publication bias exhibited significant asymmetry with statistical significance (Begg’s test, *z* = 3.59, *P*<0.001). The pooled HR was 2.37 (95% CI: 2.03–2.75) after the trim and fill analysis (Fig 3A). Sensitively analysis was further conducted with significant heterogeneity (*I*^*2*^ = 72%, *P*<0.00001, HR 2.43, 95%CI: 1.76–3.36) for multivariate analysis of SUV_max_ and DFS (2279 patients) (Fig 2B) to estimate the effect of each study on the pooled HR by omitting one study at a time. Three studies [34, 44, 55] were omitted, and an HR of 3.24 (2.43–4.33) using a fixed-model was obtained with a decreased I^2^ of 38% and a P value of 0.07 in the Q test. The Begg’s test was statistically significant (*z* = 2.23, *P* = 0.026). The pooled HR was 1.77 (95% CI: 1.29–2.43) after the trim and fill analysis (Fig 3B).

Five studies (881 patients) indicated that a larger MTV predicted worse local control using univariate analysis with a pooled HR of 2.27 (95% CI: 1.77–2.90, *P* = 0.16, *I*^*2*^ = 39%) (Fig 2C). Three studies obtained the same result [26, 27, 52] for multivariate analysis, and 1 study reported undetermined results [28]. The combined HR of all 4 studies (782 patients) was 2.49 (95% CI: 1.23–5.04, *P* = 0.04, *I*^*2*^ = 63%) (Fig 2D). High TLG was associated with poor local control in 4 studies (821 patients) using univariate analysis with a combined HR of 2.46 (95% CI: 1.91–3.17, *P* = 0.39, *I*^*2*^ = 1%) (Fig 2E). Multivariate analysis with 3 studies (730 patients) revealed a combined HR of 2.97 (95%CI: 1.68–5.28, *P* = 0.09, *I*^*2*^ = 59%) (Fig 2F).

### Secondary Outcome: OS

**Fig 4:**
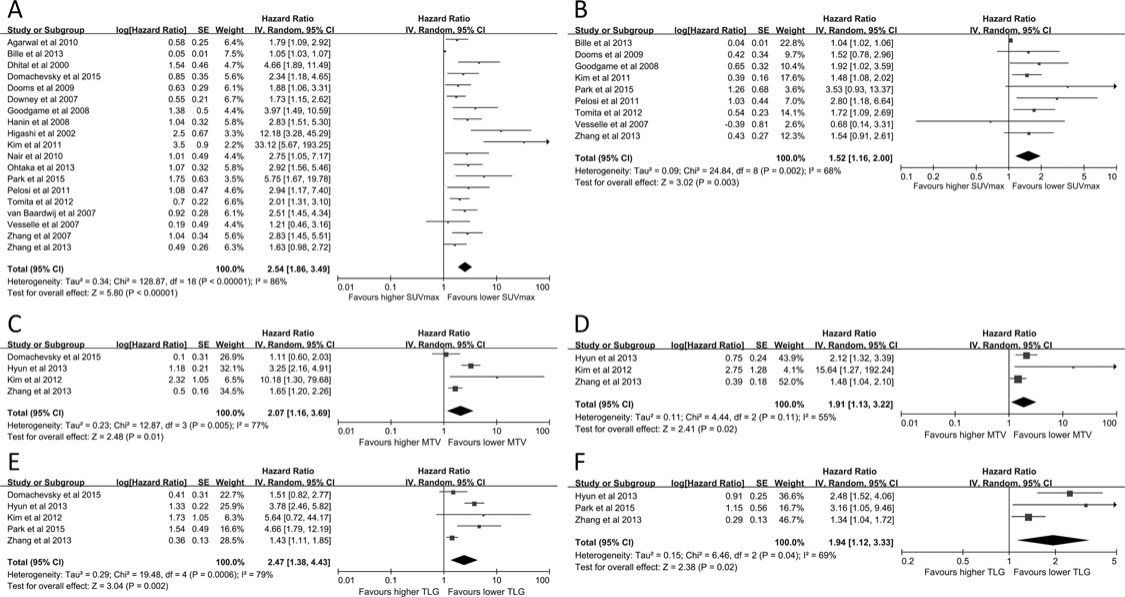
Forest plots of HR for deaths with SUV_max_ (A, unadjusted; B, adjusted), MTV (C, unadjusted; D, adjusted) and TLG (E, unadjusted; F, adjusted). The Chi^2^ test is a measurement of heterogeneity. *P*<0.05 indicates significant heterogeneity. Squares = individual study point estimates. Horizontal lines = 95% CIs. Rhombus = summarized estimate and its 95%CI. Fixed: fixed effect model. Random: random effect model.

Univariate analysis of 19 studies (3178 patients) explored the prognostic role of SUV_max_ for OS and demonstrated a combined HR of 2.54 (95%CI: 1.86–3.49, *P*<0.00001, *I*^*2*^ = 86%) (Fig 4A). *I*^*2*^ was not statistically significant (17 studies, 2698 patients, *P* = 0.19, *I*^*2*^ = 23%) after the exclusion of two studies [34, 39] with an HR of 2.26 (95%CI: 1.94–2.64). Begg’s test revealed no significant publication bias (*z* = 1.47, *P* = 0.141). Heterogeneity also existed (*I*^*2*^ = 68%, *P* = 0.002, HR = 1.52, 95%CI: 1.16–2.00) in adjusted analyses of SUV_max_ and OS rate (9 studies, 1467 patients) (Fig 4B). Exclusion of the report from Bille et al.[39] reduced this heterogeneity and led to a *P* value of 0.69 (8 studies, 1063 patients, *I*^*2*^ = 0%). A fixed-effect model revealed that the combined HR reached 1.64 (95%CI: 1.34–1.99).

Larger MTV predicted poor OS in univariate and multivariate analyses. Significant disparities were determined in unadjusted (4 studies, 860 patients, HR 2.07, 95%CI: 1.16–3.69, *P* = 0.005, *I*^*2*^ = 77%) and adjusted analyses (3 studies, 679 patients, HR = 1.91, 95%CI: 1.13–3.22, *P* = 0.11, *I*^*2*^ = 55%) (Fig 4C and 4D). High TLG was associated with poor OS in univariate analysis (5 studies, 1108 patients) with a combined HR of 2.47 (95%CI: 1.38–4.43, *P* = 0.0006, *I*^*2*^ = 79%). Multivariate analysis of 3 studies with 836 patients also demonstrated a significant prognostic role of TLG for OS (HR 1.94, 95%CI: 1.12–3.33, *P* = 0.04, *I*^*2*^ = 69%) (Fig 4E and 4F).

### Stratified Analyses of SUV_max_

Table 3 summarizes total and stratified results. The combined HRs of SUV_max_ for stage I in the sub-group analysis for DFS according to disease stage were 3.62 (95%CI: 2.72–4.81, *P* = 0.07, *I*^*2*^ = 41%) from univariate analysis and 3.35 (95%CI: 2.18–5.16, *P* = 0.46, *I*^*2*^ = 0%) from multivariate analysis. Publication bias existed (Z = 2.81, P = 0.005) in univariate analysis, and the pooled HR was 3.00 (95%CI: 2.30–3.91) after the trim and fill process (Fig 3C). The combined HRs of SUV_max_ from univariate analysis of stage I and stage II for OS were 3.43 (95%CI: 1.75–6.75, *P* = 0.01, *I*^*2*^ = 66%) and 2.64 (95%CI: 1.11–6.31, *P* = 0.23, *I*^*2*^ = 31%), respectively.

**Table 3:** Total and subgroup analyses of SUV_max_, MTV and TLG in surgical NSCLC.

Endpoint	Parameter	Factor	Data source	No. of studies	HR	95%CI of HR	Heterogeneity, *I*^*2*^(%)	Model used
DFS	SUV_max_	Total	Unadjusted	22	2.74	2.33–3.24	32	Fixed
			Adjusted	17	2.43	1.76–3.36	72	Random
		Stage I	Unadjusted	11	3.62	2.72–4.81	41	Fixed
			Adjusted	6	3.35	2.18–5.16	0	Fixed
		ADC	Unadjusted	4	4.81	2.87–8.08	47	Fixed
			Adjusted	4	2.92	1.19–7.17	77	Random
		Non-ADC	Unadjusted	3	1.98	1.04–3.79	0	Fixed
		Surgery only	Unadjusted	19	2.75	2.30–3.29	41	Fixed
			Adjusted	15	2.30	1.65–3.20	72	Random
		Threshold ≤ 5.9	Unadjusted	14	3.02	2.42–3.77	46	Fixed
			Adjusted	11	4.63	2.53–8.48	62	Random
		Threshold > 5.9	Unadjusted	10	2.42	1.89–3.11	38	Fixed
			Adjusted	7	1.68	1.07–2.63	58	Random
	MTV	Total	Unadjusted	5	2.27	1.77–2.90	39	Fixed
			Adjusted	4	2.49	1.23–5.04	63	Random
	TLG	Total	Unadjusted	4	2.46	1.91–3.17	1	Fixed
			Adjusted	3	2.97	1.68–5.28	59	Random
OS	SUV_max_	Total	Unadjusted	19	2.54	1.86–3.49	86	Random
			Adjusted	9	1.52	1.16–2.00	68	Random
		Stage I	Unadjusted	6	3.43	1.75–6.75	66	Random
			Adjusted	2	2.14	1.21–3.77	0	Fixed
		Stage II	Unadjusted	3	2.64	1.11–6.31	31	Fixed
		Surgery only	Unadjusted	16	2.27	1.93–2.66	48	Fixed
			Adjusted	7	1.59	1.30–1.95	0	Fixed
		Threshold ≤ 5.9	Unadjusted	8	3.47	2.10–5.71	68	Random
			Adjusted	3	1.61	1.22–2.12	0	Fixed
		Threshold > 5.9	Unadjusted	10	2.12	1.44–3.13	85	Random
			Adjusted	5	1.42	0.97–2.08	67	Random
	MTV	Total	Unadjusted	4	2.07	1.16–3.69	77	Random
			Adjusted	3	1.91	1.13–3.22	55	Random
	TLG	Total	Unadjusted	5	2.47	1.38–4.43	79	Random
			Adjusted	3	1.94	1.12–3.33	69	Random

**Abbreviations:** SUV_max_: maximal standardized uptake value; MTV: metabolic tumor volume; TLG: total lesion glycolysis; NSCLC: non-small cell lung cancer; HR: hazard ratio; CI: confidence interval; DFS: disease-free survival; OS: overall survival; ADC: adenocarcinoma.

Sub-group analysis based on the histology type revealed that the combined HRs of SUV_max_ on DFS from univariate analysis for adenocarcinoma and non-adenocarcinoma were 4.81 (95%CI: 2.87–8.08, *P* = 0.13, *I*^*2*^ = 47%) and 1.98 (95%CI: 1.04–3.79, *P* = 0.64, *I*^*2*^ = 0%), respectively. Four studies provided multivariate analysis of SUV_max_ on DFS for adenocarcinoma patients, with a pooled HR of 2.92 (95%CI: 1.19–7.17, *P* = 0.005, *I*^*2*^ = 77%).

The combined HRs of SUV_max_ on DFS were 2.75 (95%CI: 2.30–3.29, *P* = 0.03, *I*^*2*^ = 41%) and 2.30 (95%CI: 1.65–3.20, *P*<0.01, *I*^*2*^ = 72%) in unadjusted and adjusted analysis, respectively, when analyses were narrowed to surgical only patients without adjuvant therapy. The pooled HRs of SUV_max_ in univariate and multivariate analysis were 2.27 (95%CI: 1.93–2.66, *P* = 0.02, *I*^*2*^ = 48%) and 1.59 (95%CI: 1.30–1.95, *P* = 0.79, *I*^*2*^ = 0%), respectively, for OS.

Cut-off values of SUV_max_ in each individual study were determined to be high (>5.9) or low (< = 5.9) based on the median value. Subgroup analyses demonstrated that the combined HRs of SUV_max_ for high cut-off value were 2.42 (95%CI: 1.89–3.11, *P* = 0.10, *I*^*2*^ = 38%) from univariate analysis and 1.68 (95%CI: 1.07–2.63, *P* = 0.03, *I*^*2*^ = 58%) from multivariate analysis. The low cut-off value studies demonstrated that the combined HRs of SUV_max_ (univariate analysis: HR 3.02, 95%CI: 2.42–3.77, *P* = 0.03, *I*^*2*^ = 46%; multivariate analysis: HR 4.63, 95%CI: 2.53–8.48, *P* = 0.003, *I*^*2*^ = 62%) were larger than the high cut-off value subgroup. Analysis of OS data also revealed a similar trend with pooled HRs of 3.47 (95%CI: 2.10–5.71, *P* = 0.003, *I*^*2*^ = 68%) and 1.61 (95%CI: 1.22–2.12, *P* = 0.38, *I*^*2*^ = 0%) for high cut-off group and 2.12 (95%CI: 1.44–3.13, *P*<0.01, *I*^*2*^ = 85%) and 1.42 (95%CI: 0.97–2.08, *P* = 0.02, *I*^*2*^ = 67%) for low cut-off group in unadjusted and adjusted analyses, respectively.

## Discussion

There is a high risk of local relapse and distant metastasis after curative resection for early-stage and localized NSCLC. Therefore, adjuvant therapy was explored to eliminate occult metastases and/or loco-regional residual tumor cells with a consequent reduction on recurrence and prolonged survival. It is essential to identify prognostic factors that may predict patients who are at a high risk of recurrence who will attain the most benefit from the adjuvant therapy to optimize the treatment. The evidence-based use of adjuvant therapy is highly dependent on clinical-pathological tumor staging information in the clinical setting. The role of ^18^F-FDG PET/CT imaging for the prediction of local control and OS in surgical NSCLC must be investigated because it may provide important biological information beyond TNM staging. The present systemic review and meta-analysis found that higher values of SUV_max_, MTV and TLG predicted a higher risk of disease recurrence or death in patients with surgical NSCLC. The positive association remained statistically significant across stratified analyses according to stage, pathology and cut-off values. FDG PET/CT may be used to select patients who are at high risk of tumor recurrence or death and may benefit from subsequent more aggressive treatments.

SUV_max_ is the most commonly used parameter in ^18^F-FDG PET/CT diagnosis and response monitoring because of high reproducibility and availability. The potential prognostic value of SUV_max_ for primary lung cancer was widely reported in various staged and treated populations [8–10, 14] (Table 4). Therefore, our meta-analysis focused on surgical NSCLC only and provided the most comprehensive information for the total population and sub-groups based on disease stage, pathological classification and cut-off values. However, SUV_max_ only provides information about a single volumetric pixel within the tumor, and it does not measure the volume or heterogeneity of metabolically active disease. Volumetric parameters, such as MTV and TLG, were investigated recently. The prognostic role of MTV and TLG was meta-analyzed in NSCLC patients with different stages [11]. Similar results were derived in our study, which focused on surgical NSCLC patients. Volume-based parameters exhibit advantages in the measurement of metabolic tumor burden, but controversy on the most appropriate segmentation method to measure MTV and TLG remains. Potential preferable performance of volumetric parameters to SUV_max_ as prognostic factors were reported by the studies [24, 28, 29, 52, 53] that reported complete data of FDG PET/CT-derived parameters. The present meta-analysis demonstrated that SUV_max_ performed equally with volumetric parameters based on existing data because of the limited data of volumetric parameters compared with FDG uptake. Other FDG PET/CT imaging characteristics beyond traditional parameters were also studied, such as intratumor FDG uptake heterogeneity. This parameter, as an area under the curve (AUC) of the cumulative histogram, and texture analysis predict tumor control [59] and are independent prognostic factors for survival [60–62] in NSCLC. However, these reports were not included in present meta-analysis because the study population was relatively small.

**Table 4:** Previous meta-analyses of 18F-FDG PET/CT on survival of NSCLC patients.

Study	Year	Stage	Treatment	No. of studies	No. of patients	Endpoints	PET parameters	HR (95% CI)
Na et al. [8]	2014	I-IV	Radiotherapy	13	1081	OS/LC	SUV_max_	OS: 1.05 (1.02–1.08) LC: 1.26 (1.05–1.52)
Nair et al. [9]	2009	I	Surgery	9	1166	OS/DFS	SUV_max_/SUV_mean_	NA
Paesmans et al. [10]	2010	I-IV	Any	24	2638	OS/DFS	SUV	2.08 (1.69–2.56)
Im et al. [11]	2014	I-IV	Any	13	1581	OS/EFS	MTV	OS: 2.31 (1.54–3.47) EFS: 2.71 (1.82–4.02)
							TLG	OS: 2.43 (1.89–3.11) EFS: 2.35 (1.91–2.89)
Berghmans et al. [14]	2008	I-IV	Any	13	1474	OS	SUV_max_	2.27 (1.70–3.02)

**Abbreviations:** FDG: fluorodeoxyglucose; PET/CT: positron emission tomography/computed tomography; NSCLC: non-small cell lung cancer; HR: hazard ratio; OS: overall survival; LC: local control; SUV_max_: maximal standardized uptake value; SUV_mean_: mean standardized uptake value; NA: not available; MTV: metabolic tumor volume; TLG: total lesion glycolysis; DFS: disease-free survival; EFS: event-free survival.

Patient heterogeneity, statistical data mining, retrospective cohorts, PET acquisition and calculations of SUV_max_ are significant contributors to heterogeneity, which limited the application of glucose uptake as a companion diagnostic/prognostic marker. NSCLC is a heterogeneous disease. Patients with different histological types, stages, surgical procedures and adjuvant treatments were included in the meta-analysis. For example, Higashi et al. [41] and Stiles et al. [44] applied similar thresholds for FDG uptake. Significant differences were found in Higashi’s study in DFS (HR 8.17, 95%CI: 2.83–23.53), but statistically significant differences in DFS were not found in Stiles’s study (HR 1.54, 95%CI: 0.92–2.56). There were more patients with stage I NSCLC (80.7% versus 76.8%) and more patients with bronchioloalveolar cell carcinoma (22.8% of BAC versus <8.3%) in Higashi’s study, which may explain the lower risk of recurrence in patients with low tumor FDG uptake. The heterogeneity in PET imaging thresholds was also obvious between the studies, which be explained by many factors, including the type of PET machine, the algorithms for iteration and reconstruction, the time elapsed between FDG injection and emission scan, and the method for threshold determination. Differences in defining the regions of interest [63] and timing of the data acquisition [64] may also result in different absolute SUV estimates.

Heterogeneity between the included reports was the main limitation of this meta-analysis. Non-English articles were excluded. The fact that small sample studies with negative results are less frequently published or published with simple descriptions led to the phenomenon of increased standard error for higher HRs. The trim and fill sensitivity analysis in the present study, which incorporates the hypothetical missing studies, did not change the general result, which suggests that the association was convincible. Individual HRs from small sample studies weighed less in the total HR, and it was also helpful to ensure the reliability of results. MTV and TLG were measured in 7 studies only. Multivariate analyses were based on 5 studies for MTV and 4 for TLG. Too little data were available to meta-analyze the values of volumetric PET/CT parameters for the prediction of patient’s prognosis. Only 2 included studies were prospectively designed, but PET as a biomarker to prognosticate or predict the response to therapy was assessed over 10 years. The prospectively designed studies [65, 66] that were ineligible for the present meta-analysis also reported primarily positive results on various FDG PET/CT-derived parameters of lung cancer patients. Our meta-analysis offers a considerably valid conclusion for clinical practice under the circumstance of insufficient evidence from prospectively designed data.

In summary, this meta-analysis demonstrated that high values of SUV_max_ and MTV derived from the pretreatment of ^18^F-FDG PET/CT predicted a higher risk of recurrence or death in surgical NSCLC patients. Our findings suggest that FDG PET/CT may be used for risk stratification in disease control and survival. Patients with tumors who exhibit intense FDG uptake may be considered at a high risk of treatment failure and may benefit from more aggressive treatment. Further individual patient data should be meta-analyzed to determine the optimal threshold for PET imaging parameters.

## Supporting Information

S1 PRISMA Checklist PRISMA checklist. (DOC)

S1 File The quality scale used in this study. (DOCX)
