# Supplementary material for: Prognostic Value of 18F-FDG PET/CT in Surgical Non-Small Cell Lung Cancer: A Meta-Analysis
Source: PLoS One. 2016 Jan 4;11(1):e0146195. doi: 10.1371/journal.pone.0146195 (PMC4699812; doi:10.1371/journal.pone.0146195)
Supplement: S1 File — (DOCX) [file pone.0146195.s002.docx]

**S1 File: The quality scale used in this study**

Except when specified, the attributed value per item is 2 points if it is clearly defined in the article, 1 point if its description is incomplete or unclear, and 0 points if it is not defined or is inadequate.

Scientific design

(1) Study objective definition. (2) Study design: prospective, 2 points; retrospective or retrolective, 1point; not defined, 0 points. (3) Outcome definition. (4) Statistical considerations: fully reported with a preliminary assessment of the patient/sample number to be included and/or analyzed, 2 points; patient/sample number to be included and/or analyzed justified by the number of studied variables (minimum 10 patients per variable), 1 point; not defined, 0 points. (5) Statistical methods and tests description.

Generalizability

(1) Patient selection criteria, including histological type, disease stage and treatment. (2) Patients’ characteristics, including histology type, disease stage and treatment. (3) Initial workup. (4) Treatment description. (5) Number of ineligible patients with exclusion causes.

Results analysis

(1) Follow-up description, including the number of events. (2) Survival analysis according to the SUV, MTV and/or TLG. (3)Univariate analysis of the prognostic factors for survival: report of the relative risk with the CI, 2 points; results without evaluation of the relative risk and its CI, 1 point; not reported or inadequate, 0 points. (4) Multivariate analysis of the prognostic factors for survival: report the relative risk with the CI, 2 points; results without evaluation of the relative risk and its CI, 1 point; not reported or inadequate, 0 points. (5) Survival curves clearly enough to be analyzed.

The PET reports

(1) Patients characteristics: weight/height; blood sugar level; histological subtype. (2)^18^F-FDG-PET acquisition protocol characteristics: fasting duration; injected dose of^18^F-FDG; delay between injection and data acquisition.(3) Technical parameters: investigation area; delay between CT thorax and PET acquisition; SUV formula; type of SUV; type of PET engine; duration of emission time; duration of transmission time; attenuation; and reconstruction parameters. (4) The analysis of the relationship between SUV was performed without knowledge of survival results and conversely (double blind). (5) SUV cut-off definition.
